# Management of Hepatitis B Virus Infection in Liver Transplantation Setting; The Rising Concerns and Growing Hopes, Report From 10th Congress of the Iranian Society for Organ Transplantation, 2011, Shiraz, Iran

**DOI:** 10.5812/hepatmon.8094

**Published:** 2012-12-30

**Authors:** Seyed Moayed Alavian, Kamran B. Lankarani, Mario Rizzetto, Alfredo Marzano, Mohsen Moghadami, Saman Nik-Eghbolian, Amin Bahrani

**Affiliations:** 1 Baqiyatallah Research Center for Gastroenterology and Liver Disease, Baqiyatallah University of Medical Sciences, Tehran, IR Iran; 2 Health Policy Research Center, Shiraz University of Medical Sciences, Shiraz, IR Iran; 3 Department of Gastroenterology, Molinette University of Torino, Torino, Italy; 4 Division of Gastroenterology and Hepatology, San Giovanni Battista Hospital, Turin, Italy; 5 HIV/AIDS Research Center, Shiraz University of Medical Sciences, Shiraz, IR Iran; 6 Shiraz Organ Transplant Unit, Namazee Hospital, Shiraz University of Medical Sciences, Shiraz, IR Iran

**Keywords:** Liver Transplantation, Hepatitis B Virus, Hepatitis B Hyperimmune Globulin, Liver Cirrhosis, Iran

## Abstract

Hepatitis B infection is the main cause of liver related mortality in many countries including Iran. Liver transplantation in cirrhosis due to HBV infection before 1990 was an absolute contraindication. Recurrent infection was a significant event in post liver transplant setting and resulted in increased risk of graft failure and death except successful transplanted individuals. Advances in antiviral prophylaxis have now made graft reinfection majority patients as a rare event. Graft and patient survival have been improved significantly during the past two decades, and consequences of transplantation for hepatitis B virus are now superior to those achieved for most other indications. This has encouraged many centers including the major liver transplantation center of Iran, in Shiraz, to provide liver transplantation to more patients with HBV related end stage liver disease. Management of these patients begins before transplantation along with special care after transplantation. There are some myths and doubts in the management of these patients and one should always balance the cost and efficiency. One of the major concerns is the high economic and social cost of recurrence and all possible efforts should be performed to avoid the ominous consequences of reinfection. Having a clear scientific grasp on the management of HBV cirrhosis before and after liver transplantation, options and protocols, and changing the concept which HBV infected are contraindicated ones for liver transplantation, and future hopes in increasing patients survival after liver transplantation using the new nucleosides analogues and availability of hepatitis B immunoglobulin in the transplantation setting. This scientific report paper outlines the insights communicated at the HBV and liver transplantation symposium during 10th Congress of the Iranian Society for Organ Transplantation, May 2011, Shiraz, Iran.

## 1. Background

Despite the dramatic decrease in prevalence of hepatitis B virus (HBV) infection because of HBV vaccination and improvement in treatment outcomes, this infection is still a major cause of end stage liver disease and subsequent liver transplantation in the world (1, 2). Before 1990, because of high rate of HBV recurrence and lower survival of patients infected with HBV, HBV infection was considered a contraindication for liver transplantation (1). Immunoprophylaxis with hepatitis B immunoglobulin (HBIG) and oral anti-HBV therapy have improved the outcome of patients with HBV infection undergone transplantation (3). Before using HBIG and oral antiviral drugs (Nucleoside analogues), recurrence of HBV infection after transplantation was the rule and it often led to liver failure and in some cases death except successful transplanted patients (4). Liver graft and patient survival in patients with HBV infection have improved significantly during the past decade and much of this progress is due to advances in antiviral prophylaxis. However there is still room for improvement in the preventive measures to avoid recurrence of HBV infection in patients undergoing liver transplantation (LT) Moreover, there is some controversy on the dosage and duration of HBIG therapy in these patients, along with efficiency and adequacy. There is currently no consensus standardized recommendation over the therapeutic options which include HBIG applications (5) and nucleoside analogues therapy (NAT). These and other issues need to be addressed in an evidence based manner. There is also need for further studies especially with newer oral antiviral drugs (6). About a decade ago with recognition of protective ability of long-term, high-dose HBIG, HBV became an accepted indication for LT leading to more patients undergone transplantation in the world and in our region (7). However, HBIG is expensive and its monotherapy use carries a risk of HBV recurrence of 15% to25% (3). There are many controversies regarding the administration route, dosage, total amount, frequency, and duration of HBIG treatment. The issue is even more complex in patients receiving concomitant oral antiviral drugs (8-11). The transition from combined HBIG and NAT treatment to NAT alone reduces the costs, side effects, and inconveniences which are associated with HBIG the frequent injections of HBIG, probably without sacrificing efficacy. This report outlines the insights communicated at the HBV and liver transplantation symposium during 10th Congress of the Iranian Society for Organ Transplantation, May 2011, Shiraz, Iran.

## 2. Hepatitis B Infection Burden in the World and the Region; the Rising Concerns and Growing Hopes for Liver Transplantation in Patients With HBV Infection

HBV infection is a major global health problem with an estimated 350 million people infected with this virus worldwide (12) and approximately 500,000 deaths annually (2). The most common etiology of liver-related death is HBV infection in many parts of the world especially in middle and far east Asia regions (2), but because of the fear of HBV infection recurrence after liver transplantation, most centers previously excluded these patients from the liver transplantation list. With adaptation of the prophylactic strategies against HBV reinfection which are now considered essential for patients who undergo liver transplantation, more patients are now receiving liver transplantation for this indication. In Iran, about 26.5% of all transplanted cases had positive results of HBs Ag test and HBV is not a contraindication for liver transplantation anymore (13).

## 3. Management of HBV Infection in Patients With Cirrhosis Before Liver Transplantation

Patients with chronic HBV infection are prone to progress to cirrhosis and complications such as portal hypertension, variceal bleeding, ascites, jaundice, encephalopathy, liver failure and hepatocellular carcinoma. In these patients the goal is identification and treatment of complications associated with end stage liver disease in the preoperative period. One should not only suppress viral replication which may improve liver function, but also diagnose and treat correctable complications. In the presence of irreversible liver disease and a life expectancy below 12 months with no effective medical or surgical alternatives to transplantation and a poor quality of life, liver transplantation should be considered. Patients with HBV-decompensated cirrhosis should be referred for LT, based on their Model of End Stage Liver Disease (MELD) score. This score encompasses certain laboratory tests and is designed to prioritize livers to those patients who are in the most need. Patients with the highest MELD scores are allocated liver for transplant in priority. The necessary medical assessments in these patients are listed in Sidebar. As the risk of developing hepatocellular carcinoma is high for patients with HBV cirrhosis, surveillance should continue even if the virus is effectively controlled with antiviral drugs (14).

**Sidebar:** The Medical Assessment Before Liver Transplantation

**A complete laboratory assessment including**
Urine analysis
Complete blood count
Blood chemistries
Liver function tests
Blood and tissue typing
Blood tests for HBV, HCV, HIV, EBV and CMV
**Several X-ray examinations, including**
Chest X-ray- to tell if the lungs are healthy.
Ultrasound with Doppler- checks the blood flow into and out of the liver looking at the liver's veins and arteries, as well as the bile ducts.
CT scan- a computerized image of the abdomen, particularly to determine the size of the liver, major blood vessels, and the presence of any tumors.
MRI (magnetic resonance imaging)- may be used in place of a CT scan.
**Electrocardiogram (EKG)- echocardiogram **
**Lung (pulmonary) function tests- to reveal how well the lungs are working. A blood sample may be taken from the patient's wrist to help determine the amount of oxygen in the blood.**
**Upper endoscopy for varices, ulcers, inflammation, or any other disease **
**Kidney tests**
**Miscellaneous tests may be necessary, such as: Pap smear and mammogram for women, PSA blood test for men to detect cancer of the prostate, dental X-rays to detect cavities or infections, and a stool test for hidden blood, which may indicate intestinal bleeding.**
**The Psychosocial Assessment**

The most important achievements in increasing the survival of patients with HBV end stage liver disease was the introduction of potent antiviral drugs. Oral therapy with nucleoside analogues for HBV infection in decompensated cirrhosis is highly effective in suppressing HBV DNA and clearance of HBV DNA from the serum of patients before LT and may result in significant clinical improvement allowing patients to be removed from the waiting list (15). Lamivudine has been shown to be very efficacious in treatment of hepatitis B in patients with decompensated cirrhosis and improvement of liver failure, but is aggravated by a high incidence of virologic breakthrough due to resistance, up to 53% in patients with cirrhosis after 48 months, with the risk of rapid decompensation and even death due to the virologic breakthrough (16). Thus, resistance to lamivudine severely limits its use in patients with HBV-related decompensated cirrhosis. Recent studies have shown that entecavir is efficacious in suppression of HBV DNA and improvement in liver failure (17). A few small studies have also shown encouraging preliminary results with tenofovir in HBV patients with decompensated cirrhosis (18, 19). We emphasize the importance of using potent antiviral agents like tenofovir or entecavir along with referral for transplantation in patients with detectable HBV DNA, regardless of HBeAg status. The choice depends on cost and previous NAT treatment. In Iran currently tenofovir is available with a much lower cost in generic form. Entecavir should not be used in patients previously treated with lamivudine because of the high risk of transresistance. We discourage the use of lamivudine in this setting. Patients with high serum HBV DNA levels regardless of their HBe Ag status and patients with antiviral drug-resistance prior to transplant are considered at high-risk of post-transplant HBV recurrence (20). In conclusion, viral suppression induced by antivirals results in a clinical improvement which may allow liver transplantation to be delayed or even avoided. We recommend starting these drugs as soon as possible in patients with decompensated cirrhosis with any detectable DNA level. Tenefovir and entecavir as monotherapy are recommended for long-life to suppress viral replication and delay the need for transplantation and to reduce the risk of HBV recurrence on the graft.

## 4. Hepatitis B Virus Recurrence after LT, Importance of HBIG Prophylaxis

Historically, LT for patients with chronic HBV infection has been associated with aggressive reinfection and poor survival results (21). LT outcomes have been improved with the routine administration of HBIG and oral antiviral agents after 1990 (21). The impact of viral load at the time of transplantation on reinfection was confirmed in multicentric studies in Europe (7) with a high rate of HBV infection recurrence in those who had detectable levels of HBV DNA at the time of transplantation and lowest recurrence rate in those with null HBV DNA nor hepatitis B e antigen detectable in their sera (7). With the use of anti-HBs immune globulin, the risk of HBV recurrence decreased significantly. In a multivariate analysis the predictors of a lower risk of HBV recurrence were the long-term administration of immune globulins, hepatitis delta virus superinfection, and acute liver failure (7). Absence of viral replication at the time of transplantation and long-term immunoprophylaxis were associated with a reduced risk of recurrent HBV infection and mortality (7). Monotherapy with HBIG can prevent reinfection of grafts after LT (5) and there is evidence for a dose-dependent response to HBIG treatment (7). High doses of HBIG (10,000 IU) monotherapy protocols have been used during the anhepatic phase, followed by 10,000 IU daily during the initial 6 days of postoperative period, .Thereafter the dosage should be titrated to reach and maintain level of anti-HBs at 100 IU/l. There is some evidence that maintaining higher titers of anti HBs at 300-500 IU /ml may be of benefit in the early post transplantation period i.e. the first year. HBIG should be continued according to serum anti-HBs titers either for short term (6 to 12 months) or indefinitely (22-26). Although some studies have shown that HBIG monotherapy appeared to be equivalent to combination therapy (HBIG plus antiviral NA) for prevention of post-LT HBV, most of the available evidence indicates clearly that combination therapy is more effective to reduce the reinfection rate (27, 28). Long-term HBIG monotherapy was generally well tolerated, although mild to moderate adverse events were reported, whereas they are rare (28, 29). However, because of the risk of late reinfection, especially in patients with active pre-LT replication of HBV (and particularly those with a viral load greater than 20,000 iu/ml), HBIG immunoprophylaxis alone might not be the ultimate solution (7, 26, 30-32). Furthermore, monotherapy with HBIG has been shown to promote mutations in the surface genes which may lead to a reduction of the efficacy of HBIG (31, 33, 34). Therefore, to overcome this problem, combination therapy of HBIG with NA was introduced and accepted as a more effective and standard approach of therapy to reduce the reinfection rate (7). The prognosis of liver transplantation in patients with HBV is related to the efficacy of prophylaxis of HBV graft reinfection (35). The risk of HBV reinfection is directly related to the HBV viral load at the time of transplantation (7). Patients who receive liver transplantation for chronic hepatitis B infection require long-term combination therapy with HBIG and oral antiviral agents to prevent graft reinfection (36). Oral antiviral agents such as lamivudine, adefovir, entecavir or tenefovir can control HBV replication in patients with decompensated HBV induced cirrhosis awaiting liver transplantation (7). The main risk in the use of these drugs is emergence of resistance and breakthrough during therapy. Thus the administration of lamivudine and adefovir alone as a prophylaxis after LT is probably insufficient particularly in patients with replicative status (37). Introduction of new and potent antiviral drugs have increased the hope for more efficient and safer suppression of HBV infection before and after liver transplantation. The current survival of patients with HBV cirrhosis after liver transplantation is more than 85% and it motivates the transplantation centers to accept more patients with HBV infection in their daily practice (35). The threshold of HBV DNA, which defines replicating status in patients undergoing liver transplantation, has remained unclear. A linear correlation was observed between recurrence and viral load at the time of operation. In transplant patients with HBV DNA higher than 20,000/mL, less than 20,000 iu/ml, and DNA undetectable by amplified assay, hepatitis B recurrence observed in 50%, 7.5%, and 0% of patients, respectively. Overall, a viral load higher than 20,000 iu/mL at the time of liver transplantation portends a high risk of hepatitis B recurrence (26). In conclusion, for prophylaxis of HBV reinfection after liver transplantation, a combination protocol of oral antiviral drugs (NA) prior to liver transplantation, and a long term antiviral NA and IV or IM HBIG following transplantation is recommended. This combination protocol is highly effective with a reduction of the rate of HBV reinfection from more than 90% to less than 10% (35). One of the most important issues in the post transplantation care of the patients is when to stop HBIG regimen. There are contradictory data on this issue (37). Considering the high cost of the drug, it is very important to determine which patient can stop HBIG without increasing the risk of infection recurrence with subsequent risk of graft loss and even mortality. There are encouraging reports of stopping HBIG in stable patients (at least one year from LT), especially in those with no viral replication at the time of operation (low risk group), however data is few and long follow-up is limited. The possibility of HBV recurrence after cessation of HBIG and possible serious outcomes should be considered in any strategy. Persistence of HBV DNA in serum, liver and peripheral blood mononuclear cells in 50% of HBV transplanted patients who have negative results of HBsAg test on HBIG long-term administration at 10 years posttransplant warns us to be more careful to stop the HBIG after LT (38). The most prudential long-term treatment remains prophylaxis with one or more antivirals and low dosages of intramuscular HBIG (1,000 IU/monthly?). This strategy is highly effective, well tolerated and with an acceptable cost.

## 5. Hepatocellular Carcinoma Is a Risk Factor for Recurrence After LT

The role of hepatocellular carcinoma (HCC) in HBV recurrence has been remained a debate. In a study by Faria et al. (39) fourteen patients (14.1%) developed HBV recurrence within a median period of 15 months post-LT. HCC in explant, a pre-LT HBV DNA viral load > or = 20,000 iu/mL, and HBIG mono-prophylaxis were independently associated with the risk of HBV recurrence post-OLT. The association of HCC recurrence with HBV recurrence in post-LT patients, and detection of HBV DNA and cDNA in HCC tissue suggest that HBV replication in tumor cells may be contributed to HBV recurrence in post-LT patients (39).

## 6. Resistance to Anti-Viral Drugs Before and After Liver Transplantation

Patients from the Mediterranean area, with negative results of HBeAg and positive results of antibodies to HBeAg (anti-HBe), were reported to have chronic hepatitis with replicating HBV. In 1989 the molecular basis of this form of HBV was discovered with the identification of HBV mutations preventing HBeAg formation from an otherwise normally replicating HBV. The most commonly studied mutation associated with this, were in the pre-core region at nucleotide (nt) 1896 where adenine (A) is substituted with guanine (G), producing a stop codon which prematurely terminates synthesis of the HBeAg. The core promoter region regulates transcription of the precore region. Therefore certain mutations in this region can affect HBeAg synthesis. Specifically, a double mutation involving substitution of T with A at nucleotide 1762 and A for G at nucleotide 1764 can reduce precore mRNA and HBeAg production (40). In patients undergoing liver transplantation for HBV-related cirrhosis, the accumulated mutations in the precore and core regions of the HBV genome may be associated with development of severe recurrent disease post-LT (41). We would like to emphasize again the impact of viral load of HBV and risk of recurrence without considering the HBeAg status in LT patients (7, 42). Lamivudine has an excellent initial therapeutic effects in suppression of HBV infection before and after LT, and preventing graft reinfection after LT (43), but selection and emergence of specific mutations in polymerase gene compromise this initial effect on HBV replication (44). A major concern has been the emergence of viral resistance to mutation in the YMDD locus of the HBV DNA polymerase gene rendering HBV less sensitive to the lamivudine (44, 45). After LT, mutants have been recognized in patients who had received lamivudine as prophylactic therapy before and after transplantation (44). Transplant centers hesitate to perform LT in patients with liver cirrhosis who develop the YMDD mutant because of concerns of graft reinfection, but passive immunoprophylaxis with high dose of HBIG is believed to neutralize HBV and prevent reinfection of transplanted liver (46). Despite HBIG and oral antiviral combination (NA) therapy, some patients develop recurrent HBV infection after liver transplantation (47). In these patients, HBV recurrence is supposed to be due to consequence of HBV overproduction from extrahepatic sites (48) or formation of virus mutants with an altered HBs Ag structure and emergence of a mutation in the pre S/S genome which renders it less susceptible to the neutralizing effects of HBIG (49). Approximately 50% of the treatment failures which occur with HBIG prophylaxis are due to mutations in the 'a' determinant of the HBV (46). It has been suggested that the emergence of escape mutations was more frequent in patients receiving HBIG manufactured from vaccines than from patients with post-HBV infection (50). Escape mutation can reverse when HBIG is stopped (49). In patients without mutations, failure of HBIG therapy may relate to the frequency and dosage of HBIG, the type and amount of immunosuppression, and the pretransplant replication status.

## 7. Liver Transplantation in Patients Coinfected With HDV and HBV

Hepatitis D virus (HDV) coinfected with HBV is most often associated with a severe and progressive liver disease leading to cirrhosis more rapidly than hepatitis B alone and are more resistant to standard antiviral drugs (51). HDV infection has an inhibitory effect on the HBV replication and most of patients with HDV/HBV coinfection, have negative results of HBV DNA test at the time of liver transplantation (52). It seems that the overall rate of HBV infection recurrence and reappearance of HBs Ag in patients with coinfection of HBV/HDV is lower than HBV mono-infected even in patients who have not received long-term HBIG (37, 53)., No reappearance of HBs Ag in serum and no recurrence of hepatitis B in the liver transplant were reported in patients with dual infection, who have received HBIG and lamivudine in follow-up for three years. In contrast to HBV recurrence rate, HDV reinfection is frequent in patients with coinfection of HBV and HDV after liver transplantation (54), but it is less severe than HBV recurrence alone (7) and if the HBs Ag test result remains negative with HBIG and nucleosides analogues after liver transplantation, the quantity of HDV Ag in the liver graft would be low and HDV markers would disappear from the liver and serum (55). In conclusion, the risk of HBV recurrence after liver transplantation in patients with coinfection of HBV and HDV when using long-term HBIG and lamivudine is very low and these patients have the best prognosis among HBV infected patients.

## 8. Liver Transplantation in HBV/HIV Coinfection

Coinfection with the HIV and the hepatitis B virus (HBV) presents a significant challenge to health care providers, with different prevalence rates in different parts of the world and in average involving approximately 10% of persons with HIV infection (56). Introduction of effective combined antiretroviral therapy has made HIV infection as a chronic illness and there are now an increasing number of HIV infected patients with HBV related end stage liver disease (57). HIV infection worsens the course of chronic HBV, resulting in faster progression of fibrosis, faster development of cirrhosis and hepatocellular carcinoma, a lower rate of spontaneous HBe or HBs sero-conversion and a greater risk of HBV reactivation in inactive carriers (56). HIV disease is no longer considered an absolute contraindication for liver transplantation (LT) in most transplant centers worldwide (58).

## 9. OBI and Transplants

Occult hepatitis B virus (OHB) is defined as the detection of HBV deoxyribonucleic acid (DNA) in the serum or liver tissue of individuals who are serologically negative for hepatitis B surface antigen (HBsAg). There is a need for a very sensitive real-time polymerase chain reaction assay to test serum HBV DNA (59). In the presence of serum hepatitis B core antibody (anti-HBc), an immunosuppressed state because of liver transplantation has been shown to induce viral reactivation in patients with occult HBV, with appearance of the typical serological profile seen in overt HBV infection (60). Occult HBV infection is common among patients with cryptogenic liver disease in Iran and in some reports more than 50% of these patients had positive results of HBV DNA with sensitive tests. These patients need to be monitored for possible reactivation of occult infection if they have anti HBc Ab and are submitted to chemotherapy (59). Another issue related to OHB after liver transplantation is liver grafts from donors with positive results of anti-HBc test which may be used for transplantation in patients with hepatitis B virus (HBV)-related liver disease, and an occult HBV infection may develop from any source. A phylogenetic analysis of the isolated HBV DNA sequence revealed HBV infection of both donor and recipient origins. Occult HBV infection after liver transplantation can originate from both the donor and recipient despite prolonged nucleoside analogue prophylaxis. The presence of intrahepatic HBV cccDNA may be attributed to a donor with previous exposure (61). The competition between recipient HBV and donor HBV may exist, and the dominant HBV strain may be determined by the replication efficiency or HBV DNA level of the strain (61). Unfortunately despite prolonged nucleoside analogue prophylaxis, intrahepatic HBV cccDNA persists and occult HBV infection is common. Therefore it is mandatory to receive lifelong antiviral prophylaxis to eliminate the risk of reactivation of HBV infection (61). In conclusion, apparent acquisition of HBV infection after liver transplantation may be related to occult pretransplant infection in the recipient or occult infection in the donor. The posttransplant outcome of this infection tends to be benign but our findings do underscore the clinical relevance of HBV infection in the absence of serological markers (60).

## 10. Iranian Experience Regarding Liver Transplantation in Patients With HBV Infection

Shiraz Organ Transplant Center in southern Iran began to perform liver transplantation since 1993, Potential recipients are placed on a waiting list based on the established standard indications for liver transplantation (62). Patients with decompensated cirrhosis with any level of HBV DNA are advised to receive antiviral therapy; in most cases with tenofovir 300 mg per day The intention is to suppress HBV DNA to undetectable levels (50 IU/ml). In rare patients on other antiviral drugs such as adefovir, if HBV DNA is negative, then the same drug is continued. At the time of transplantation HBIG 10000 unit is administered intravenously immediately in the anhepatic phase of operation then 5000 unit/day IV in two divided doses for 7 days and after that according to serum titer of anti HBs Ab. The target is Anti HBs Ab titer of 500IU/ml in the first month, 300IU/ml in months 2 to 6 and thereafter 100IU/ml, Usually the required dose is 1250 unit per day or every other day in the first month, reduced to 1250 IU every week to every other week in the first 6 month followed by 1250 IU monthly. Except for the first two weeks in which the drug is used intravenously, intramuscular injection is the preferred route. At the same time patients receive antiviral therapy with tenofovir 300 mg daily. Dose adjustment according to renal function is performed in those with GFR less than 10%. We recommend continuation of HBIG combined with NA indefinitely, although we had several patients who discontinued HBIG after the first year mainly due to economic reasons with no recurrence, although most of these patients were from low risk group with negative results of HBV DNA test in pretransplantation evaluation. All patients should be monitored with HBs Ag and Anti HBs Ab titers. Anti HBs Ab should be checked just before receiving the next dosage. This is mandatory to have the nadir of Anti HBs Ab titer not the maximum. In rare patients with abnormal results of liver function test and negative results of HBs Ag test, if other causes of raised liver enzymes is ruled out then HBV DNA would be checked. Otherwise in postoperative period HBV DNA is not checked routinely. With this protocol the 1 year survival for these patients is now 83.4% and the recurrence rate of HBV in grafts was rare (Un-published data).

## 11. Conclusion

In management of patients with cirrhosis and HBV infection, we recommend starting antiviral therapy as soon as possible in the presence of any level of HBV DNA. Normal level of aminotransferases cannot exclude the patients from antiviral therapy. The goal of therapy is to decrease the HBV DNA level to undetectable status. The best strategy for patients with cirrhosis before liver transplantation is Tenofovir for naïve cases and periodic monitoring of HBV DNA viral load. In cases that have given Lamivudine for a long time, we have two strategies, the first is to follow up by periodic monitoring for HBV DNA viral load at least every six months and after deterioration, adding Tenofovir is recommended. The second approach is adding Tenofovir if HBV DNA viral load is not available and switching to Tenofovir in those with less severe liver disease after at least 3 months of combination therapy. In posttransplant period, we should divide the status to early and long-term follow up. The best strategy is to start HBIG in the anhepatic phase with a dose of 10, 0000 IU intravenously, then 5000 IU daily for the first week and reduce to 2500 daily in the second week, and finally adjust according to the level of anti-HBs Ab. If the result of anti-HBs level is not immediately available, we recommend the prescription of fixed dosage of HBIG. We start NA according to the pretransplantation state as soon as oral feeding is possible. In patients with severe renal failure the dose of antiviral therapy should be adjusted. In long-term follow up, we use HBIG indefinitely currently. Periodic monitoring of Anti-HBs Ab and HBs Ag are recommended at least every three months. Most cases of HBV reinfection occurred during the first three years posttransplantation and rarely thereafter (7). The patients with S mutants are managed with increasing dosage of HBIG but this has been a rare event in our center. Patients with coinfection of HBV/HDV viruses have a better course. The management is similar to HBV mono-infected ones and if HBV DNA viral load is high, oral antiviral nucleosides is recommended. Liver transplantation in patients with cirrhosis and coinfection of HIV/HBV is still experimental and it is not recommended in those with CD4 < 100/μl. Patients with HBV infection complicated by HCC should be scheduled for transplantation according to the specific criteria for HCC. The risk of recurrence of HBV infection is higher in these patients and they should be monitored periodically with HBV DNA viral load and alpha-fetoprotein level.
